# Characterization and potential functional significance of human-chimpanzee large INDEL variation

**DOI:** 10.1186/1759-8753-2-13

**Published:** 2011-10-25

**Authors:** Nalini Polavarapu, Gaurav Arora, Vinay K Mittal, John F McDonald

**Affiliations:** 1 Parker H Petit Institute for Bioengineering & Bioscience, School of Biology, Georgia Institute of Technology, 315 Ferst Drive NW, Atlanta, GA 30332, USA; 2 Monsanto Co, 800 North Lindberg Boulevard, St Louis, MO 63146, USA

**Keywords:** insertion and deletion, differential gene expression, retrotransposon, noninterspersed sequence, human insertion, short interspersed nuclear element

## Abstract

**Background:**

Although humans and chimpanzees have accumulated significant differences in a number of phenotypic traits since diverging from a common ancestor about six million years ago, their genomes are more than 98.5% identical at protein-coding loci. This modest degree of nucleotide divergence is not sufficient to explain the extensive phenotypic differences between the two species. It has been hypothesized that the genetic basis of the phenotypic differences lies at the level of gene regulation and is associated with the extensive insertion and deletion (INDEL) variation between the two species. To test the hypothesis that large INDELs (80 to 12,000 bp) may have contributed significantly to differences in gene regulation between the two species, we categorized human-chimpanzee INDEL variation mapping in or around genes and determined whether this variation is significantly correlated with previously determined differences in gene expression.

**Results:**

Extensive, large INDEL variation exists between the human and chimpanzee genomes. This variation is primarily attributable to retrotransposon insertions within the human lineage. There is a significant correlation between differences in gene expression and large human-chimpanzee INDEL variation mapping in genes or in proximity to them.

**Conclusions:**

The results presented herein are consistent with the hypothesis that large INDELs, particularly those associated with retrotransposons, have played a significant role in human-chimpanzee regulatory evolution.

## Background

Although humans and chimpanzees have accumulated significant differences in a number of phenotypic traits since diverging from a common ancestor about six to eight million years ago, their genomes are more than 98.5% identical at protein-coding loci [1]. Since this modest degree of nucleotide divergence does not seem sufficient to explain the extensive phenotypic differences that exist between the two species, it has been hypothesized that the genetic basis of the differences lies at the level of gene regulation [2] and is associated with the extensive insertion and deletion (INDEL) variation between the two species [3].

A number of comparative genomic studies focused on specific chromosomal regions of humans and nonhuman primates that have been carried out have revealed that significant INDEL variation exists between these species [4,5]. For example, in a comparison of human chromosome 21 and the syntenic chimpanzee chromosome 22, as many as 68,000 INDELs were identified [6]. We have shown previously that interspersed repeats, particularly retrotransposons (RTs), have contributed significantly to the INDEL variation between humans and chimpanzees [7]. Because RT sequences located in or near genes have the capacity to significantly alter patterns of gene expression, it has long been recognized that these elements may be important factors in regulatory evolution [8-16]. Other sources of INDEL variation between chimpanzees and humans are simple tandem repeats (TRs) and other noninterspersed sequences (NISs) [17]. Because NISs in or near genes are capable of altering gene expression, they also have been postulated to play a role in regulatory evolution [18-23].

In this article, we present our detailed characterization of large INDEL variation (80 to 12,000 bp in length) associated with human and chimpanzee genes and test if this variation is significantly correlated with differences in gene expression in a variety of tissues. We characterize INDELs by type (that is, chimpanzee insertion (CI), chimpanzee deletion (CD), human insertion (HI) and human deletion (HD) of interspersed sequences and/or NISs). Our results indicate that both interspersed repeats (predominately RTs) and NISs have contributed significantly to human-chimpanzee genome evolution, primarily due to insertions within the human lineage. This variation is significantly correlated with previously determined differences in gene expression consistent with the hypothesis that large INDEL variation has played a significant role in human-chimpanzee evolution.

## Results and discussion

**Figure 1:**
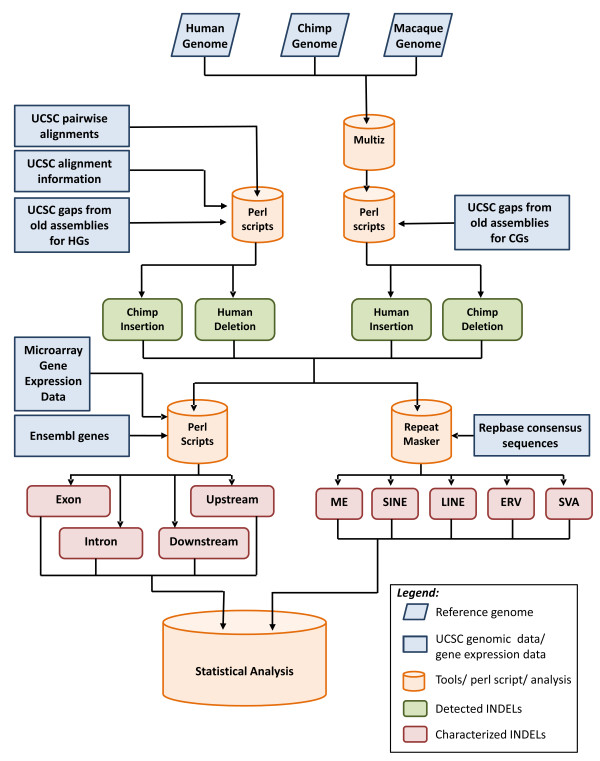
**Computational pipeline for the detection and characterization of human and chimpanzee insertions and deletions**. Using information from the designated databases, we characterized insertions and deletions (INDELs) and analyzed them using various in-house Perl scripts and open source algorithms (Multiz, RepeatMasker [44] and Tandem Repeats Finder [45]). The multiple alignment program Multiz was used to classify chimpanzee gaps (CGs) as insertions or deletions. The UCSC Genome Browser [40] pairwise alignment databases were used for human gap (HG) classification as insertions or deletions. Human and chimpanzee INDELs were associated with the known human and chimpanzee Ensembl genes [30] obtained from the UCSC Table Browser (http://genome.ucsc.edu/cgi-bin/hgTables), and the presence of INDELs was correlated with the microarray gene expression data. INDEL sequences that were obtained from their corresponding reference genomes were searched for various repeat elements using RepeatMasker and Tandem Repeats Finder and classified according to the families of repeat sequences (partial or complete) present within each INDEL. The characterized INDELs were then assessed using various statistical analytical methods.

The computational pipeline of our analysis is outlined in Figure 1 (see Methods for additional information).

### Characterization of human and chimpanzee gaps

We use the term "human gaps" (HGs) to refer to sequences present in chimpanzees but absent in humans and the term "chimpanzee gaps" (CGs) for sequences present in humans but absent in chimpanzees [7]. Collectively, these gaps constitute the INDEL variation (defined in this study as gaps ranging in size from 80 to 12,000 bp) between humans and chimpanzees. Using the database available at the UCSC Genome Bioinformatics web site [24], we identified a total of 26,509 INDELs (11,365 HGs and 15,144 CGs) (Table 1). The majority (18,574/26,509, or 70%) of these INDELs are interspersed sequences associated with transposable elements (TEs) (that is, complete, truncated or composite TE sequences repeated multiple times throughout the genome). Nearly all the TE-associated INDELs (18,476/18,574, or 99.5%) are homologous to RT sequences. The 30% (7,935/26,509) of INDELs not associated with TEs are composed of what we refer to as "noninterspersed sequences," or NISs, the majority of which (5,335/7,935, or 67%) are gaps of unique sequence (US) (that is, sequences uniquely associated with a single INDEL). The remainder of the NISs (2,600/7,935, or 33%) is composed of TRs (Table 1).

**Table 1:** Number of INDELs associated with different categories of sequences

Categories of gaps	Human gaps	Chimpanzee gaps	Total INDELs (HGs + CGs)
Total gaps	11,365	15,144	26,509
Interspersed repeats (all)	7,176	11,398	18,574
Interspersed sequences (retrotransposons)	7,121	11,355	18,476
Retrotransposons (SINEs)	3,494	7,021	10,515
Retrotransposons (LINEs)	1,847	2,052	3,899
Retrotransposons (ERVs)	519	356	875
Retrotransposons (SVAs)	114	681	795
Retrotransposons (MEs)	1,147	1,245	2,392
Interspersed sequences (DNA elements)	55	43	98
Noninterspersed sequences (all)	4,189	3,746	7,935
Noninterspersed sequences/tandem repeats (NIS/TR)	1,266	1,334	2,600
Noninterspersed sequences/unique sequences (NIS/US)	2,923	2,412	5,335

CG = chimpanzee gap; ERV = endogenous retrovirus; HG = human gap; INDEL = insertion and deletion; LINE = long interspersed nuclear element; ME = mosaic element; NIS = noninterspersed sequence; SINE = short interspersed nuclear element; SVA = biologically active composite elements consisting of fragments of SINE, VNTRs and Alu elements; TR = tandem repeat; US = unique sequence; VNTR = variable number of tandem repeats. Interspersed repeats are transposable element sequences that are present multiple times throughout the genome. The majority of interspersed repeats are retrotransposon sequences (subcategories: SINEs, LINEs, ERVs, SVAs, and MEs). DNA family transposable elements constitute less than 1% of interspersed repeats. Noninterspersed sequences are TRs or USs that map to specific INDEL sites in the genome.

### The majority of human-chimpanzee INDELs are a result of insertions

The presence of a sequence in humans (or *vice versa *in chimpanzees) that is missing at an orthologous genomic position in chimpanzees (humans) can be due to either an insertion in one species or a deletion in the other. Since DNA TEs compose less than 0.4% (98/26,509, or 0.37%) of human-chimpanzee INDEL variation, the following analysis of the relative contribution of insertions and deletions is limited to RTs and NISs.

By using Rhesus macaques (*Macaca mulatta*) as an out-group, we determined that 63% (16,518/26,411) of the INDEL variation between humans and chimpanzees is due to insertions (Table 2). The vast majority of all insertions are associated with RTs (12,683/16,518, or 77%), and the majority of RT insertions have occurred in the human lineage (8,648/12,683, or 68%) (Table 3). Indeed, 61% ((5,399 + 10,607)/26,411) of all human-chimpanzee INDELs can be attributed to events (insertions or deletions) that occurred within the human lineage after the two species diverged from a common ancestor (Table 2). The percentage of all insertions and deletions that occurred in the human lineage that are associated with RTs is 64% ((3,086 + 8,648)/18,476) (Table 3), and the percentage associated with NIS insertions and deletions is 54% ((2,313 + 1,959)/7,935) (Table 4). In contrast to RT-associated INDELs, where insertions clearly predominate (12,683/18,476, or 69%) (Table 3), NIS-associated INDELs are more equally attributable to insertion (3,835/7,935, or 48%) and deletion (4,100/7,935, or 52%) events (Table 4).

**Table 2:** Number of human and chimpanzee INDELs associated with all sequences (retrotransposons and noninterspersed sequences)

All sequences	Human gaps			Chimpanzee gaps			Total insertions	Total deletions	Total INDELs
Retrotransposons + noninterspersed sequences	CIs	HDs	CIs + HDs	HIs	CDs	HIs + CDs	CIs + HIs	CDs + HDs	CIs + HIs + CDs + HDs
Total	5,911	5,399	11,310	10,607	4,494	15,101	16,518	9,893	26,411

CD = chimpanzee deletion; CI = chimpanzee insertion; HD = human deletion; HI = human insertion; INDEL = insertion and deletion. Using Rhesus macaque as an out-group, we characterized INDEL variation as CDs, CIs, HDs or HIs.

**Table 3:** Number of human and chimpanzee INDELs associated with retrotransposons

	Human gaps			Chimpanzee gaps					
				
Retrotransposon subclass	CIs	HDs	CIs + HDs	HIs	CDs	HIs + CDs	Total insertions (CIs + HIs)	Total deletions (CDs + HDs)	Total INDELs (CIs + HIs + CDs + HDs)
SINE	2,264	1,230	3,494	5,787	1,234	7,021	8,051	2,464	10,515
LINE	1,311	536	1,847	1,756	296	2,052	3,067	832	3,899
ERV	208	311	519	156	200	356	364	511	875
SVA	98	16	114	680	1	681	778	17	795
ME	154	993	1,147	269	976	1,245	423	1,969	2,392
Total	4,035	3,086	7,121	8,648	2,707	11,355	12,683	5,793	18,476

CD = chimpanzee deletion; CI = chimpanzee insertion; ERV = endogenous retrovirus; HD = human deletion; HI = human insertion; INDEL = insertion and deletion; LINE = long interspersed nuclear element; ME = mosaic element; SINE = short interspersed nuclear element; SVA = biologically active composite elements consisting of fragments of SINE, VNTRs and Alu elements. Using Rhesus macaque as an out-group, we characterized INDEL variation as CDs, CIs, HDs or HIs.

**Table 4:** Number of human and chimpanzee INDELs associated with noninterspersed sequences

	Human gaps			Chimpanzee gaps					
				
Noninterspersed sequence subclass	CIs	HDs	CIs + HDs	HIs	CDs	HIs + CDs	Total insertions (CIs + HIs)	Total deletions (CDs + HDs)	Total INDELs (CIs + HIs + CDs + HDs)
TRs	720	546	1,266	814	520	1,334	1,534	1,066	2,600
USs	1,156	1,767	2,923	1,145	1,267	2,412	2,301	3,034	5,335
Total	1,876	2,313	4,189	1,959	1,787	3,746	3,835	4,100	7,935

CD = chimpanzee deletion; CI = chimpanzee insertion; HD = human deletion; HI = human insertion; INDEL = insertion and deletion; TR = tandem repeat; US = unique sequence. Using Rhesus macaque as an out-group, we characterized INDEL variation as CDs, CIs, HDs or HIs.

We grouped INDELs associated with RTs into five groups based upon the subclass of RTs associated with each INDEL: (1) short interspersed nuclear elements (SINEs), (2) long interspersed nuclear elements (LINEs), (3) endogenous retroviruses (ERVs), (4) biologically active composite elements consisting of fragments of SINEs, VNTRs (variable number of tandem repeats), and Alu elements (SVAs) and (5) "mosaic elements" (MEs), a term we will use to refer to inactive sequences composed of a mosaic of more than one class of the above-named RT homologous sequences. Of the RTs associated with HGs, 49% (3,494/7,121) are homologous to SINEs, 26% (1,847/7,121) to LINEs, 7% (519/7,121) to ERVs, 2% (114/7,121) to SVAs and 16% (1,147/7,121) to MEs (Table 1). Of the RTs associated with CGs, 62% (7,021/11,355) are homologous to SINEs, 18% (2,052/11,355) to LINEs, 3% (356/11,355) to ERVs, 6% (681/11,355) to SVAs and 11% (1,245/11,355) to MEs (Table 1). These values are proportionate to the relative frequency of the various classes of RTs in the human and chimpanzee genomes [1,25].

Consistent with the relative transpositional activity of RT families in humans and chimpanzees [1,25], we found that the majority of the RT-associated insertions involve SINEs and LINEs (Table 3). RTs with low or undetectable transpositional activity (ERVs and SVAs) were rarely associated with insertions. We found that the frequency of ERV insertions is 1.3-fold higher in chimpanzees than in humans (208/156 = 1.3-fold) (Table 3), predominately due to the expansion of two chimpanzee-specific endogenous retrovirus families (CERV 1/PTERV 1 and CERV 2) three to five million years ago [7,26,27]. In contrast, we found that the frequency of SVA-associated insertions is 6.9-fold higher in humans than in chimpanzees (680/98 = 6.9-fold) (Table 3), which is consistent with the presence of transpositionally active SVA subfamilies in the human lineage [28,29]. Overall, we found that the frequency of RT-associated insertions is more than twofold higher in humans than in chimpanzees (8,648/4,035 = 2.1-fold). The frequency of LINE-associated, SVA-associated and ERV-associated deletions is, on average, higher in humans than in chimpanzees, whereas the frequency of SINE-associated and ME-associated deletions is nearly the same in both species (Table 3).

As stated above, we grouped INDELs associated with NISs into two classes: those associated with TRs and those not associated with TRs that we classify as USs. We found that the majority of NIS INDELs are associated with US (5,335/7,935, or 67%), most of which (3,034/5,335, or 57%) are deletions (Table 4). In contrast, the majority of TR-associated INDELs are insertions (1,534/2,600, or 59%).

### Most INDELs located in or in proximity to human and chimpanzee genes are the consequence of retrotransposon insertions within the human lineage

Of the 34,914 human/chimpanzee genes listed in the Ensembl database (March 2006 build) [30], 10,597 (10,597/34,914, or about 30%) are associated with INDELs (that is, having one or more INDELs located in or within 5 kb upstream or downstream of a gene) (Table 5). The majority of INDELs associated with human genes are insertions (HI/HI + HD: 4,193/(4,193 + 2,034) = 67%), and the proportion of INDELs associated with chimpanzee genes is about equally distributed between insertions and deletions (CI/CI + CD: 2,125/(2,125 + 2,245) = 49%) (Table 5). The percentage of genes associated with RT-containing INDELs ((6,873 + 816)/10,597 = 73%) is more than two times greater than the percentage of genes associated with NIS-containing INDELs ((2,908 + 816)/10,597 = 35%) (Table 5). The majority of RT INDELs associated with genes is the result of insertions or deletions within the human lineage ((3,149 + 326) + (1,139 + 155)/(6,873 + 816) = 62%), and the vast majority of these events are due to insertions ((3,149 + 326)/((3,149 + 326) + (1,139 + 155) = 73%) (Table 5). In contrast, the frequencies of NIS INDELs associated with genes are more nearly equal within the human lineage (((718 + 326) + (740 + 155))/(2908 + 816) = 52%) and the chimpanzee lineage (((674 + 175) + (776 +160))/(2,908 + 816) = 48%). Similarly, the overall frequencies of NIS insertions (((718 + 326) + (674 + 175))/((2,908 + 816)) = 51%) and deletions (((740 + 155) + (776 + 160))/(2,908 + 816) = 49%) are more nearly the same.

**Table 5:** Number of genes associated with different types of INDELs

Type of INDEL	Genes associated with INDELS containing RTs only	Genes associated with INDELs containing NISs only	Genes associated with INDELs containing both RTs and NISs	Total (genes associated with INDELs)
HI	3,149	718	326	4,193
CI	1,276	674	175	2,125
HD	1,139	740	155	2,034
CD	1,309	776	160	2,245
Total	6,873	2,908	816	10,597

CD = chimpanzee deletion; CI = chimpanzee insertion; HD = human deletion; HI = human insertion; INDEL = insertion and deletion; NIS = noninterspersed sequence; RT = retrotransposon sequence. The genes associated with INDELs were classified on the basis of the type of INDEL (HIs, CIs, HDs and CIs) associated with the gene and type of sequence contained in the INDEL (RTs vs NISs).

### Human-chimpanzee INDEL variation is correlated with differences in gene expression

Although the identification, quantification and characterization of human-chimpanzee INDEL (and other types of genetic) variation are relatively straightforward, the establishment of whether this variation may be of potential functional and/or adaptive significance is not. One approach taken by evolutionary biologists in addressing this question is to correlate differences in genetic variation between species with differences in levels of gene expression [31]. Such comparative studies can be problematic because the lack of a significant correlation between differences in gene expression and a specific genetic variant or class of variants at a particular life stage or from a particular tissue does not preclude the possibility that significant correlations will exist at other life stages and/or in other tissues not examined. Nevertheless, if statistically significant correlations are found at even a single life stage or in a single tissue, they can be informative and suggestive of potentially productive areas of future research.

To explore possible correlations between human-chimpanzee INDEL variation and differences in gene expression, we reanalyzed a previously published human-chimpanzee expression data set consisting of expression arrays from five different tissues (brain, testis heart, liver and kidney) [31]. A major goal of this previous study was to correlate sequence differences with expression differences and a number of microarray probe sets for which quality sequences could not be obtained in humans and chimpanzees (for example, those required for the calculation of K_a_/K_s _ratios) were excluded. Since the quality of the chimpanzee genome sequence has improved in recent years, and because our interest is in the possible contribution of INDELs to chimpanzee-human expression differences, we reanalyzed this microarray data set, including probe sets that had previously been excluded.

Of the 20,676 (Affymetrix May 2004 build) genes examined in our reanalysis, we found that 17,755 (17,755/20,676, or 86%) are expressed genes (we define "expressed genes" as those designated as "present" by default in MAS 5.0 Affymetrix software (Affymetrix Inc, Santa Clara, CA USA) in at least one tissue in either chimpanzees or humans) and that 15,004 (15,004/17,755, or 85%) of these expressed genes display a significant between-species difference (*P *< 0.05) in expression in at least one of the five tissues examined (Table 6). The most dramatic difference in gene expression between humans and chimpanzees is in testis, where 70% of expressed genes (10,803/15,445) display a significant difference in expression between chimpanzees and humans, followed by heart (51%), brain (49%), kidney (47%) and liver (39%) (Table 6).

**Table 6:** Number of genes differentially expressed between humans and chimpanzees across five tissues

	Tissue type				
	
Expressed genes	Brain	Testis	Heart	Liver	Kidney
Number of genes expressed	14,133	15,445	13,497	13,684	14,059
Number of genes differentially expressed	6,884 (49%)	10,803 (70%)	6,843 (51%)	5,308 (39%)	6,589 (47%)

ANOVA = analysis of variance. "Expressed genes" are those designated as "present" by the default Affymetrix MAS 5.0 software in at least one tissue in either chimpanzees or humans. ANOVA was used to identify genes whose expression was significantly different (*P *< 0.05) between humans and chimpanzees for each of the tissue types. The percentages in parentheses were calculated by dividing the number of genes differentially expressed or not in each tissue by the total number of genes expressed in that tissue.

Of all expressed genes (in the tissues and adult life stages examined), an average of 30% were associated with INDELs (brain: ((2,266 + 2,153)/14,133 = 31%; testis: (3,438 +1,256)/15,445 = 30%; heart: (2,233 + 1,948)/13,497 = 31%; liver: (1,696 + 2,466)/13,684 = 30%; and kidney: (2,179 + 2,144)/14,059 = 31%) (Table 7). Of differentially expressed (DE) genes, an average of 33% (brain: 2,266/(2,266 + 4,618) = 33%; testis: 3,438/(3,438 + 7,365) = 32%; heart: 2,233/(2,233 + 4,610) = 33%; liver: 1,696/(1,696 + 3,612) = 32%; and kidney: 2,179/(2,179 + 4,410) = 33%) were associated with INDELs (Table 7).

**Table 7:** Number of differentially expressed or non-differentially expressed genes associated or not associated with INDELs

Tissue type	Number of DE genes associated with INDELs	Number of non-DE genes associated with INDELs	Number of DE genes not associated with INDELs	Number of non-DE genes not associated with INDELs	Total expressed genes
Brain	2,266	2,153	4,618	5,096	14,133
Testis	3,438	1,256	7,365	3,386	15,445
Heart	2,233	1,948	4,610	4,706	13,497
Liver	1,696	2,466	3,612	5,910	13,684
Kidney	2,179	2,144	4,410	5,326	14,059

DE = differentially expressed; INDEL = insertion and deletion; kb = kilobase; non-DE = non-differentially expressed. INDELs mapping within or in proximity (± 5 kb) to genes were considered to be associated.

The proportion of DE genes associated with INDELs was significantly greater (*P *< 0.05) than the proportion of non-differentially expressed (non-DE) genes associated with INDELs in all five tissues, indicating that the association of INDELs with genes may be of functional significance (Table 8). Partitioning these differences in proportion to RT-associated INDELs and NIS-associated INDELs indicates that the functional differences are attributable to both types of INDELs, although the majority of DE genes are associated with RTs (Tables 9 and 10).

**Table 8:** Proportions of differentially expressed or non-differentially expressed genes associated with INDELs are significantly different

Tissue type	DE genes associated with INDELs/total DE genes (%)	Non-DE genes associated with INDELs/total non-DE genes (%)	Proportions test (*P*-value)
Brain	2,266/2,266 + 4,618 (33%)	2,153/2,153 + 5,096 (30%)	4.054E-05
Testis	3,438/3,438 + 7,365 (32%)	1,256/1,256 + 3,386 (27%)	3.93E-09
Heart	2,233/2,233 + 4,610 (33%)	1,948/1,948 + 4,706 (29%)	2.7E-05
Liver	1,696/1,696 + 3,612 (32%)	2,466/2,466 + 5,910 (29%)	0.0019
Kidney	2,179/2,179 + 4,410 (33%)	2,144/2,144 + 5,326 (29%)	2.35E-08

DE = differentially expressed; INDEL = insertion and deletion; non-DE = non-differentially expressed. Proportions for all INDELs across all tissues are shown.

**Table 9:** Proportions of differentially expressed or non-differentially expressed genes associated with INDELs are significantly different

Tissue type	DE genes associated with RT INDELs/total DE genes (%)	Non-DE genes associated with RT INDELs/total non-DE genes (%)	Proportions test (*P*-value)
Brain	1,916/2,266 + 4,618 (28%)	1,790/2,153 + 5,096 (25%)	2.42E-05
Testis	2,862/3,438 + 7,365 (26%)	1,072/1,256 + 3,386 (23%)	9.63E-06
Heart	1,876/2,233 + 4,610 (27%)	1,636/1,948 + 4,706 (25%)	0.00019
Liver	1,416/1,696 + 3,612 (26%)	2,072/2,466 + 5,910 (25%)	0.012
Kidney	1,843/2,179 + 4,410 (28%)	1,776/2,144 + 5,326 (24%)	1.52E-08

DE = differentially expressed; INDEL = insertion and deletion; non-DE = non-differentially expressed, RT = retrotransposon sequence. Proportions for all RT-associated INDELs across all tissues are shown.

**Table 10:** Proportions of differentially expressed or non-differentially expressed genes associated with INDELs are significantly different

Tissue type	DE genes with NIS-associated INDELs/total DE genes (%)	Non-DE genes with NIS-associated INDELs/total non-DE genes (%)	Proportions test (*P*-value)
Brain	801/2,266 + 4,618 (12%)	762/2,153 + 5,096 (1%)	0.036
Testis	1,193/3,438 + 7,365 (11%)	440/1,256 + 3,386 (0.94%)	0.0041
Heart	777/2,233 + 4,610 (11%)	658/1,948 + 4,706 (0.98%)	0.006
Liver	590/1,696 + 3,612 (11%)	838/2,466 + 5,910 (1%)	0.041
Kidney	732/2,179 + 4,410 (11%)	768/2,144 + 5,326 (1%)	0.11

DE = differentially expressed; INDEL = insertion and deletion; non-DE = non-differentially expressed; NIS = noninterspersed sequence. Proportions are shown for all NIS-associated INDELs across all tissues.

To further explore the hypothesis that INDELs may contribute to gene expression differences between chimpanzees and humans, we computed the proportion of genes associated (or not associated) with INDELs and DE relative to the proportion of genes associated (or not associated) with INDELs that were non-DE. We reasoned that if the presence or absence of an INDEL in or in proximity to chimpanzee and human genes is not a contributing factor to differences in gene expression, the proportion of genes associated (or not associated) with INDELs should be approximately equal for DE and non-DE genes. For example, of the 15,445 genes expressed in testis, 4,694 (3,438 + 1,256) were associated with INDELs and 10,751 (7,365 + 3,386) were not associated with INDELs (Table 7). Of the 4,694 expressed genes associated with INDELs, 73% (3,438/4,694) were DE genes. In contrast, of the 10,751 genes expressed in testis that were not associated with INDELs, 69% (7,365/10,751) were DE genes. These proportions are significantly different (p = 3.93E-09), which is consistent with the hypothesis that the association of genes with an INDEL is of functional significance for DE genes in testis at the life stage examined (Table 11). The same analysis was carried out for genes expressed in the other tissues, and the results indicate that the proportion of DE genes associated with INDELs is consistently higher than the proportion of DE genes not associated with INDELs (Table 11).

**Table 11:** Proportions of differentially expressed genes associated or not associated with INDELs are significantly different

Tissue type	DE genes with INDELs/total genes with INDELs (%)	DE genes with non-INDELs/total genes with non-INDELs (%)	Proportions test (*P*-value)
Brain	2,266/2,266 + 2,153 (51%)	4,618/4,618 + 5,096 (48%)	4.054E-05
Testis	3,438/3,438 + 1,256 (73%)	7,365/7,365 + 3,386 (69%)	3.93E-09
Heart	2,233/2,233 + 1,948 (53%)	4,610/4,610 + 4,706 (5%)	2.7E-05
Liver	1,696/1,696 + 2,466 (41%)	3,612/3,612 + 5,910 (38%)	0.0019
Kidney	2,179/2,179 + 2,144 (5%)	4,410/4,410 + 5,326 (45%)	2.35E-08

DE = differentially expressed; INDEL = insertion and deletion. Proportions for all INDELs across all tissues examined are shown.

### Little overlap exists between differentially expressed genes associated with INDELs and differentially expressed genes associated with nucleotide sequence differences between species

As indicated previously, the gene expression data used in our analysis were originally generated by Khaitovich *et al. *[31], and we used them to look for correlations with human-chimpanzee nucleotide variation.. We were interested in determining the degree of overlap between DE genes associated with INDEL variation identified in our study with DE genes previously associated with nucleotide variation in the Khaitovich *et al. *study.

**Figure 2:**
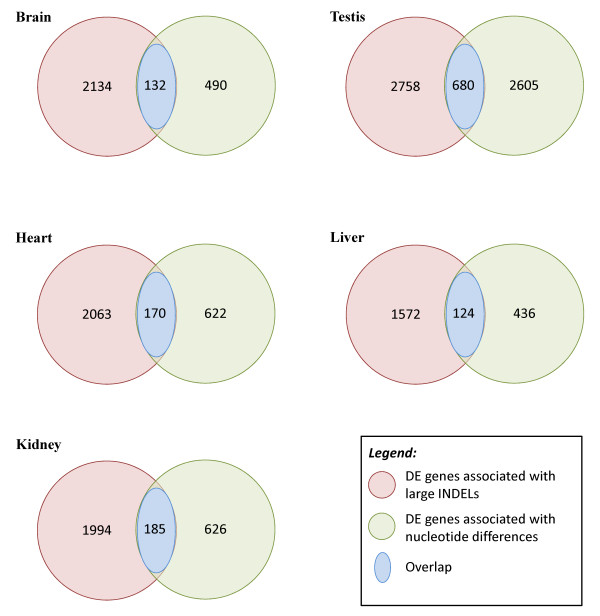
**Overlap (blue region) between genes significantly differentially expressed between humans and chimpanzees and associated with nucleotide differences (green region) **[31]**or large insertion and deletion differences (red region) between the species**. On average, fewer than 9% of genes differentially expressed at the life stages and tissues examined were associated with both types of variation. The number of differentially expressed genes associated with nucleotide differences as determined by Khaitovich *et al. *[31], as well as the number of differentially expressed genes associated with large insertions and deletions (INDELs) as determined in this study, are shown. The number of overlapping genes are shown at the intersection.

The results presented in Figure 2 indicate that, on average, fewer than 9% of the genes found to be differentially expressed between humans and chimpanzees in these two studies were associated with both nucleotide and INDEL variation. Of the 2,266 DE genes in brain and associated with INDEL variation, only 132 (132/2,266, or approximately 6%) were also associated with differences in nucleotide sequence. Similarly low proportions were found for DE genes in heart (170/2,233, or approximately 8%), liver (124/1,696, or approximately 7%) and kidney (185/2,179, or approximately 8%). Interestingly, the greatest degree of overlap was associated with DE genes in testis (680/3,438, or approximately 20%).

Testis is also the tissue where we found INDEL variation to be most highly and consistently correlated with differences in gene expression (Tables 8, 9 and 10). As previously pointed out by Khaitovich *et al. *[31], a majority of DE genes between human and chimpanzee testes are involved in reproduction and map to the X chromosome, making them potentially more responsive than autosomal loci to selection for differences in reproductive function.

## Summary and conclusions

Over the approximately six million years since the human and chimpanzee lineages diverged from a common ancestor, the two species evolved a variety of distinctive morphological, behavioral, cognitive and other phenotypic traits [32]. To explore the genetic basis of the phenotypic differences that distinguish humans from chimpanzees, a number of comparative genomic studies have been conducted in recent years [1,33]. Perhaps the most surprising finding of these studies is the paucity of protein-coding nucleotide variations between these two species, which supports earlier contention that the basis of the phenotypic differences lies in the realm of gene regulation [2].

Direct evidence in support of the regulatory hypothesis has recently been provided by a number of comparative microarray studies showing that significant differences in gene expression patterns exist between humans and chimpanzees, especially in organs (for example, brain and testis) and functions (for example, cognitive ability and fertility) directly related to some of the major phenotypic traits distinguishing the two species [31,32]. Questions remain, however, concerning the genetic basis of the differences in gene regulation that separates humans from chimpanzees. One hypothesis is that the substantial INDEL variation that exists between humans and chimpanzees may contribute significantly to the regulatory differences between the species [3,7]. In an effort to address this hypothesis, we categorized the large (80 to 12,000 bp) INDEL variation existing between humans and chimpanzees that is located in or near genes and conducted a preliminary analysis to assess whether this variation might be of functional significance. We found that 70% of the 26,509 human-chimpanzee INDELs are homologous to RT sequences (primarily SINEs and LINEs) that have inserted within the human genome subsequent to the divergence of the two species from a common ancestor. The remaining 30% of the human-chimpanzee INDEL variation is associated with US NISs or with NISs composed of TRs.

Large INDELs were found to map within or in proximity to (± 5 kb) 30% of human-chimpanzee genes. The majority of INDELs mapping within or in proximity to human genes are RT sequences, and the INDELs mapping within or in proximity to chimpanzee genes are about equally distributed between RTs and NISs. SINEs and LINEs were the most frequent categories of RTs associated with human-chimpanzee genes, which is consistent with the fact that these are the most transpositionally active classes of RTs in both species.

We found that the proportion of DE genes associated with INDELs is significantly greater than the proportion of DE genes not associated with INDELs across all tissues examined. Similarly, the proportion of DE genes associated with INDELs was significantly greater than the proportion of non-DE genes and was associated with INDELs across all tissues examined. These findings, coupled with the observation that there is relatively little overlap (fewer than 9% averaged across all tissues) between DE genes associated with nucleotide variation and those associated with large INDEL variation, are consistent with the hypothesis that large INDELs have contributed significantly to regulatory differences between humans and chimpanzees at the life stage and in the tissues examined in this study. Indeed, we have previously presented evidence that RT INDELs may have contributed to differences in apoptotic function between the two species, possibly accounting for the relatively larger size of the human brain's being pleiotropically coupled with an increased propensity for cancer development [34].

Although more extensive studies involving larger sample sizes and multiple life stages are needed to more precisely assess the relative contribution of INDELs and nucleotide differences to human-chimpanzee differences in gene expression, the preliminary analyses presented herein and previously reported by Khaitovich *et al. *[31] indicate that both classes of genetic variation contribute significantly to differences in patterns of gene expression between the two species, especially in testis.

The fact that most of the human-chimpanzee INDEL variation that correlates with differences in gene expression is attributable to HIs is interesting for two reasons. First, it is consistent with the considerable body of evidence suggesting that much of the divergence in gene expression between chimpanzees and humans may have been driven by accelerated regulatory evolution within the human lineage [35-39]. Our results are consistent with the hypothesis that an accelerated rate of INDELs (predominately RT insertions) within the human lineage may also have contributed significantly to the regulatory differences between these two species. Second, our data suggest that, at least with respect to the evolutionary contribution of INDELs to chimpanzee-human divergence in gene expression, selection operating on *de novo *mutations (for example, insertions that occurred after the divergence of the two species from a common ancestor) may have been more important than selection operating on standing INDEL variation preexisting in common ancestral populations. This second conclusion is contingent on the generally held presumption that transposition rates in humans and chimpanzees are approximately equal. Whereas previous analyses of gene expression and protein-coding sequence variation between chimpanzees and humans have revealed a pattern consistent with neutral evolution and negative selection [31], our findings are consistent with the hypothesis that INDELs in general, and RT insertions within the human lineage in particular, have been a positive driving force behind human regulatory evolution.

## Methods

### Initial data sets

Reference genome coordinates for CGs (on human genome assembly (July 2003 build)) and HGs (on panTro assembly (November 2003 build)) of sizes ranging from 80 to 12,000 bp were obtained using the UCSC Table Browser [40,41]. The CG data set was originally generated by aligning the chimpanzee genome against the human genome build hg16 (July 2003 build) and the HG data set by aligning the human genome against the chimpanzee genome build panTro1 (November 2003 build) [40,41]. The CG and HG genomic coordinates were updated to the hg18 version (March 2006 build) and the panTro2 version (March 2006 build) of the human and chimpanzee genomes, respectively, using the Batch Coordinate Conversion liftOver tool [42]. Some of the gap sequences (76 CGs and 2,581 HGs) not represented in the new versions of genome assemblies were removed in this process. Genomic sequences corresponding to the updated gap coordinates were downloaded from the UCSC Genome Database.

We derived gap coordinates from the older UCSC genome browser assemblies (hg16 (2003) and panTro1 (2003)) because these gap coordinates are not provided in the newer assemblies (hg18 (2006) and panTro2 (2006)). The gaps derived from the earlier assemblies, however, were confirmed (after converting them using the liftOver tool) in the newer assemblies by multiple and pairwise genome alignments (see Figure 1). Only those gaps that were confirmed to be present in the more recent assemblies were used in our analysis. Only regions of the human and chimpanzee genomes that could be unambiguously aligned with one other (that is, well-assembled contigs of both genome assemblies) were used in identification of the INDELs. Genomic regions containing ambiguous bases (N's) and/or assembly gaps were excluded from our analysis. HGs and CGs characterized as partial deletions or partial insertions due to incomplete sequencing of the Rhesus macaque (out-group) genome were also excluded from our analysis.

### Identification of INDELs

CGs and HGs were further categorized as INDELs by comparing reference genome alignments of the human genome (hg18), the chimpanzee genome (panTro2) and the Rhesus macaque genome (rheMac2). Reference genome sequences were obtained from the UCSC Genome Browser [43]. To identify INDELs, we followed different approaches for CGs and HGs. For CGs, the chimpanzee and Rhesus macaque genomes were aligned with the human genome to produce a three-way multiple-genome alignment. For HGs, instead of performing whole-genome multiple alignments, we consolidated pairwise alignments of human-chimpanzee, chimpanzee-Rhesus macaque and human-Rhesus macaque genomes that were already available in the UCSC Genome Browser database. Genomic coordinates of gaps were used to search the genomic regions associated with CGs and HGs in genomic alignments (multiple-genome alignment for CGs and consolidated pairwise alignment for HGs). Using the presence or absence of gap sequence in the out-group (Rhesus macaques) genome, we characterized each gap as a chimpanzee (human) deletion or human (chimpanzee) insertion. Pairwise alignment consolidation and comparison of genomic regions were done using in-house Perl scripts.

### Characterization of sequences associated with INDELs

The RepeatMasker program [44] was used to identify all interspersed repeats in the INDEL sequences. These were further classified according to the type of interspersed repeats, such as SINEs, LINEs, ERVs, SVAs or DNA elements. INDEL sequences consisting of more than one type of interspersed repeat (for example, ERVs inserted within LINE elements, etc) were classified as MEs. The Tandem Repeats Finder program [45] was used to identify TR sequences within the INDELs characterized as NISs (that is, INDELs not containing interspersed repeat sequences). The remainder of the NISs was classified as USs.

### Association of human and chimpanzee genes with the INDEL variation

The genomic coordinates of the genic regions of the human and chimpanzee Ensembl genes were downloaded from the UCSC Genome Bioinformatics website [24]. An INDEL was considered to be associated with the gene if the genomic coordinates of the INDEL mapped within or 5 kb upstream or downstream of the gene. In-house Perl scripts were used to match these coordinates.

### Microarray gene expression data analysis

The human-chimpanzee gene expression data from five different tissues (brain, heart, liver, kidney and testis) in six humans and five chimpanzees were obtained from a previous study [31]. The samples were studied using Affymetrix Human Genome U133 Plus 2.0 arrays. The expression data were reanalyzed using the following procedure. The data were processed using the MAS normalization method encoded in the Affymetrix function library of the Bioconductor package (http://www.bioconductor.org/) developed for the R statistical programming environment (http://www.r-project.org/) [45]. The genes with significant sequence differences in Affymetrix probes between humans and chimpanzees and with inconsistent hybridization patterns within samples in a species were removed. The reason for filtering is to differentiate real detection of expression in chimpanzee from expression differences due to probe mismatch, because chimpanzee expression data are derived by hybridizing to the human Affymetrix chip. The genes with detection *P*-values of less than 0.065 were considered for further analysis. The expression values of these genes were normalized across samples by *Z*-score calculation using TIBCO Spotfire DecisionSite software (http://spotfire.tibco.com/products/decisionsite.cfm; TIBCO Software, Inc, Somerville, MA, USA). Genes with *t*-test *P*-values less than 0.05 between human and chimpanzee were considered DE genes.

### Correlating INDEL variation with differential gene expression

Differences in gene expression between chimpanzee and human in each of the five tissues were partitioned for DE or non-DE genes and associated with INDELs. We looked for evidence of selection by comparing the proportion of DE genes associated with INDELs with the proportion of DE genes not associated with INDELs across all tissues examined. Similarly, we compared the proportion of DE genes associated with INDELs with the proportion of non-DE genes and associated with INDELs across all tissues examined. Proportions tests (R statistical software package [46]) were used to determine whether the differences in proportions were statistically significant (*P *< 0.05).

### Categories of genes associated with INDEL variation between humans and chimpanzees

Genes associated with HGs and CGs were analyzed in two different ways: (1) On the basis of the type of gap sequence, whether the gene is homologous to an interspersed sequence or not. For this analysis, we divided the INDEL variation data set into two different categories: (a) interspersed INDEL variation and (b) noninterspersed INDEL variation (interspersed INDEL variation was further divided into RT INDEL variation and non-RT INDEL variation); (2) On the basis of the location of the INDEL variation, that is, upstream of the transcription start site or downstream (within 5 kb downstream of the transcription termination site) of a gene. Some genes were associated with INDEL variation in two or more regions, that is, a gap starting upstream of the gene and ending at the first intron. Such genes were included in more than one category, depending on the regions covered by gap sequences. The genes associated with RT INDEL variation were further divided based on RT class and whether the sequence is homologous to SINEs, LINEs, ERVs, SVAs or MEs. As with the previous analysis, some genes were associated with many gap sequences, each of which is homologous to a different class of RT sequences. Such genes were included in more than one category, depending on the number of RT classes contained in the gap sequences.

### Linking INDEL variation with differential expression

The genes in each of the above-defined categories were checked for their expression levels between humans and chimpanzees in each of the five tissues. We used the same criteria described above in considering a gene as detected or DE between humans and chimpanzees. All genes that were detected but non-DE were considered non-DE between humans and chimpanzees. We used the R statistical software package to measure the statistical significance of the differential expression of genes associated with different categories of INDEL variation. We looked for evidence of selection by comparing the proportion of DE genes associated with INDELs with the proportion of DE genes not associated with INDELs across all tissues examined. Similarly, we compared the proportion of DE genes associated with INDELs with the proportion of non-DE genes associated with INDELs across all tissues examined. We used a proportions test to measure the statistical significance of the comparisons described above. *P *< 0.05 was considered statistically significant.

### Identification of differentially expressed genes that are correlated with both INDELs and single nucleotide variation

A list of DE genes between humans and chimpanzees in the five tissues tested (brain, testis, heart, liver and kidney) as well as those associated with single-nucleotide variation was obtained from the supplementary information published by Khaitovich *et al. *[31]. These genes were compared with DE genes (between the two species) as well as INDEL variation-associated genes that were obtained in our analyses (Additional file 1).

## Abbreviations

ANOVA: analysis of variance; bp: base pair; CD: chimpanzee deletion; CDS: coding sequence; CERV/PTERV: chimpanzee endogenous virus/*Pan troglodytes *endogenous retrovirus; CG: chimpanzee gap; CI: chimpanzee insertion; DE: differentially expressed; ERV: endogenous retrovirus; HD: human deletion; HG: human gap; hg: human genome; HI: human insertion; INDEL: insertion and deletion; kb: kilobase pair; LINE: long interspersed nuclear element; ME: mosaic element; NIS: noninterspersed sequence; non-DE: non-differentially expressed; panTro: chimpanzee genome; rheMac2: Rhesus macaque genome; RT: retrotransposon sequence; SINE: short interspersed nuclear element; SVA: biologically active composite elements consisting of fragments of SINE, VNTRs and Alu elements; TE: transposable element; TR: tandem repeat; US: unique sequence; VNTRs: variable number of tandem repeats.

## Competing interests

The authors declare that they have no competing interests.

## Authors' contributions

NP and JM conceptualized the original study. NP, GA and VM conducted the analyses. JM, NP, GA and VM wrote the manuscript. All authors read and approved the final manuscript.

## Supplementary Material

Additional file 1 **Insertion and deletion-associated genes differentially expressed between humans and chimpanzees**. Microsoft Excel file listing all insertions and deletion (INDEL)-associated genes differentially expressed between humans and chimpanzees for each tissue type (brain, testis, heart, liver and kidney). Click here for file
